# Long-term results of extended intersphincteric resection for very low rectal cancer: a retrospective study

**DOI:** 10.1186/s12893-016-0133-6

**Published:** 2016-04-18

**Authors:** Hyun Sung Kim, Sanghwa Ko, Nahm-gun Oh

**Affiliations:** Department of Surgery, Biomedical Research Institute, Pusan National University Hospital, 305 Gudeok-Ro, Seo-Gu, Busan, 602-739 South Korea

**Keywords:** Intersphincteric resection, Rectal cancer, Colonic J, Pouch–neoadjuvant chemoradiotherapy, Oncologic outcome, Fecal incontinence

## Abstract

**Background:**

Intersphincteric resection (ISR) has become an increasingly popular optional surgical tool for the treatment of very low rectal cancer. The purpose of this study was to assess the long-term oncological and functional outcomes of intersphincteric resection for T2 and T3 rectal cancer situated below 4 cm from the anal verge.

**Methods:**

A total of 62 consecutive patients with very low rectal cancer who underwent ISR from 2001 to 2010 were classified into standard ISR for T2 lesions (Group I, *n* = 24) and extended ISR for T3 lesions (Group II, *n* = 38).

**Results:**

The 5-year overall survival rates were 95.8 % for group I and 94.7 % for group II. The 5-year recurrence-free survival rates were 87.5 % for group I and 86.8 % for group II. Bowel functions were evaluated at the 12^th^ and 24^th^ months after ileostomy closure in both groups. The frequency of bowel evacuation was higher in patients who underwent extended ISR than in those who underwent standard ISR at the 12^th^ month (*p* < 0.05). However, at the 24^th^ month, the frequencies decreased in both groups, exhibiting no significant difference. In the comparison based on the Kirwan classification, group I showed better continence status than group II but no significant difference. The Wexner scores of both groups revealed that the average score was 7.33 ± 2.8 in group I and 8.18 ± 2.9 in group II at the 12^th^ month, and at the 24^th^ month, the average score was 5.21 ± 1.7 in group I and 5.82 ± 1.9 in group II. There were no statistically significant differences between the two groups.

**Conclusions:**

Extended ISR with quadrant resection of the upper external sphincter achieved good post-operative continence status, OS and RFS. Extended ISR can thus be an alternative to abdominoperineal resection for very low rectal cancer without compromising the chance of cure and improving quality of life.

## Background

Total mesorectal excision (TME) composed of complete excision of the mesorectum and securing a safe resection margin has become the standard surgical therapeutic principle in patients with low rectal cancer. From a functional point of view, preserving the quality of life by preventing damage to the autonomic nervous system and maintaining the functions of the anal sphincter, when oncologically feasible, are also important issues to be considered.

Numerous studies regarding surgical treatment of low rectal cancer have been performed over the past several decades, and the standard principle of curative surgery has been established [1–3]. Studies found that it is important to secure the distal and circumferential resection margin to prevent recurrence of the cancer. Regarding the distal resection margin in low rectal cancer, the literature approved that as short as 1 cm of the distal resection margin could be safe from an oncologic perspectives [4].

Moreover the advent of neoadjuvant chemoradiotherapy has made it possible to perform sphincter-preserving surgery by downsizing and downstaging of primary tumors. Recent advances in technical skills and surgical equipments (e.g. double stapling technique and robotic surgery using the da Vinci system) made the sphincter-preserving surgery rather a more common radical surgical procedure in low rectal cancer [5–7]. Accordingly, abdominoperineal resection (APR) was significantly reduced compared to past decades, and surgery to preserve the sphincter has improved. Thus, sphincter preservation surgery has become more common in the treatment of low rectal cancer.

An inevitable deadlock, however as ever, can be encountered when performing a sphincter-preserving procedure in very low rectal cancer less than 4 cm from anus. In these circumstances, an APR has to be still performed practically.

Schiessel et al. introduced intersphincteric resection (ISR) to extend anus-preserving procedure distally into the intersphincteric plane with removal of the internal sphincter muscle [8]. Many studies have reported favorable results in both aspects of oncologic safety and functional outcome [8–10]. Several studies have also reported treatment results from the resection of some of the external and the internal anal sphincter [11, 12].

Most studies revealed preservation of the whole external anal sphincter is indispensable for maintenance of good continence. However, it was often difficult to ensure a safe resection margin in patients with T3 and we have performed extended ISR with quadrant resection of upper external anal sphincter in these cases.

The purpose of this study was to assess the long-term oncologic and functional outcomes of extended ISR by comparing the conventional standard ISR and extended ISR in patients with very low rectal cancer less than 4 cm from the anal verge.

## Methods

### Patients

Sixty-two patients with pathologically proven rectal adenocarcinoma between January 2001 and December 2010 were selected as subjects from the database of Department of Surgery, Pusan National University Hospital. Selection criteria were patients with low rectal cancer with well or moderately differentiation located less than 4 cm from the anal verge to inferior margin of the tumor. Exclusion criteria for ISR were poorly differentiated or signet ring cell adenocarcinoma diagnosed by biopsy, suspected tumor invasion of levator ani, impaired fecal continence preoperatively. Abdominoperineal resection (APR) was performed in these patients with resectable primary tumor.

Staging work-up included digital rectal examination, colonoscopy with biopsy, abdominal CT, MRI, and anorectal manometry for performing anorectal function assessment.

Fifty-three patients with locally advanced rectal cancer (LARC) received the neoadjuvant chemoradiotherapy. It composed of total irradiation dose of 40.5–50.4 Gy delivered in conventional fractionation (daily fractions of 1.8 Gy in weekdays for over 5–6 weeks) and capecitabine or 5-fluorouracil with low-dose folinic acid as the radiosensitizer. Capecitabine was administered orally at a dose of 825 mg/m^2^ twice a day throughout the radiation therapy. Fluorouracil bolus (425 mg/m^2^) synchronously with folinic acid (20 mg/m^2^) was delivered on five consecutive days in the first and last week. For standardization of the assessment, preoperative T staging after neoadjuvant chemoradiation was determined by MRI, for the sixty-two patients as follows: Group I (yT2N0, *n* = 19; yT2N1, *n* = 5), Group II (yT3N0, *n* = 22; yT3N1, *n* = 13; yT3N2, *n* = 3).

### Surgical technique

All surgical procedures, open surgery, were performed or supervised by an experienced surgeon. For the abdominal approach, the same procedure as the low anterior resection was carried out after midline abdominal incision in modified supine position. The inferior mesenteric artery was ligated at the origin from the abdominal aorta. The splenic flexure was completely mobilized to mandate tension-free coloanal anastomosis and pelvic dissection was performed while preserving the superior hypogastric plexus. The rectum was completely dissected free from the pelvic floor and levator ani. A colonic J-pouch was made using the proximal colon after completion of abdominal phase of the procedure.

**Fig. 1:**
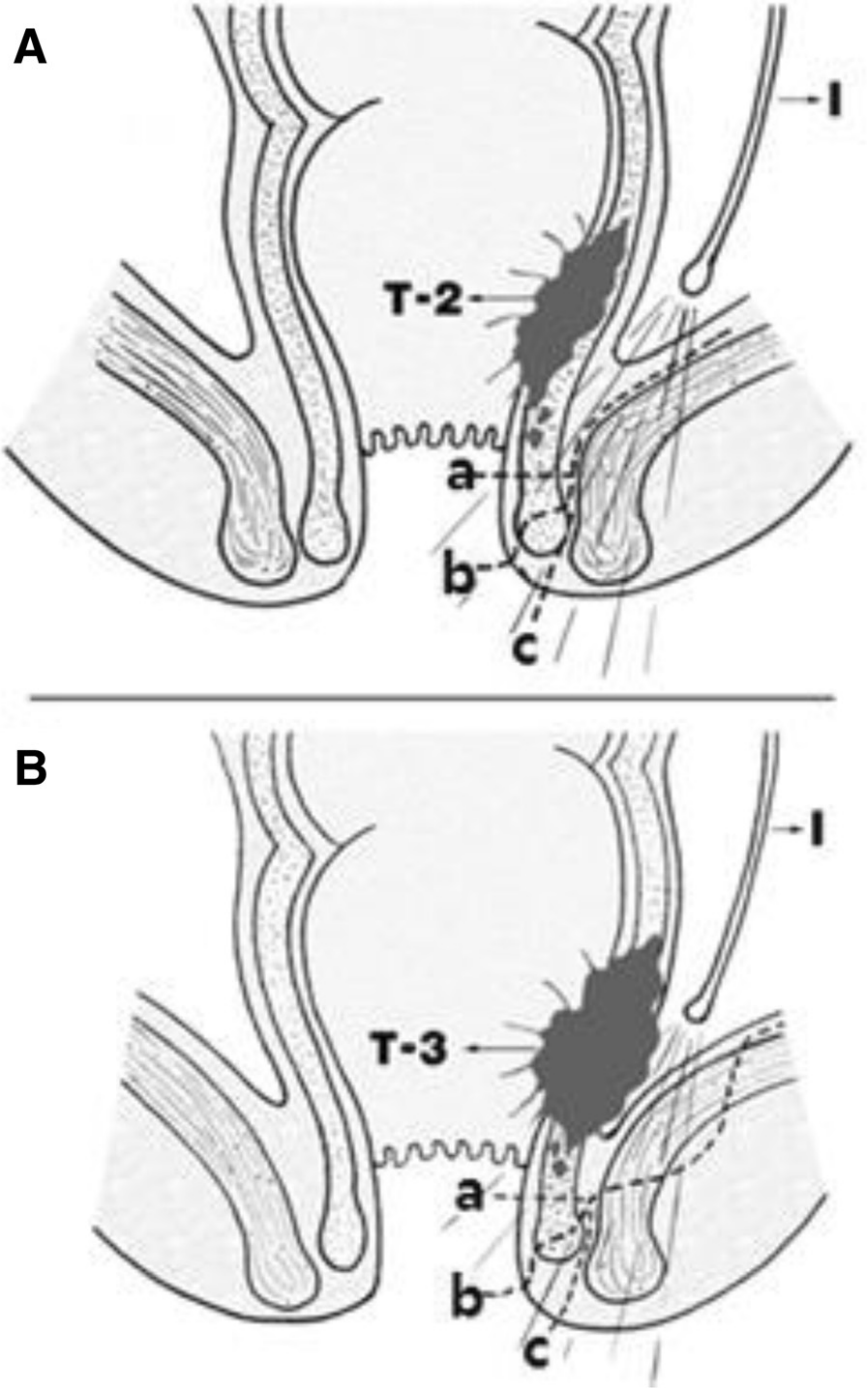
Schematic representation of two options for ISR. **a** Standard ISR for T2 tumor. **b** Extended ISR for T3 tumor. I illuminator. *a*,*b*,*c* The distal resection line of the internal sphincter depending on tumor level

**Fig. 2:**
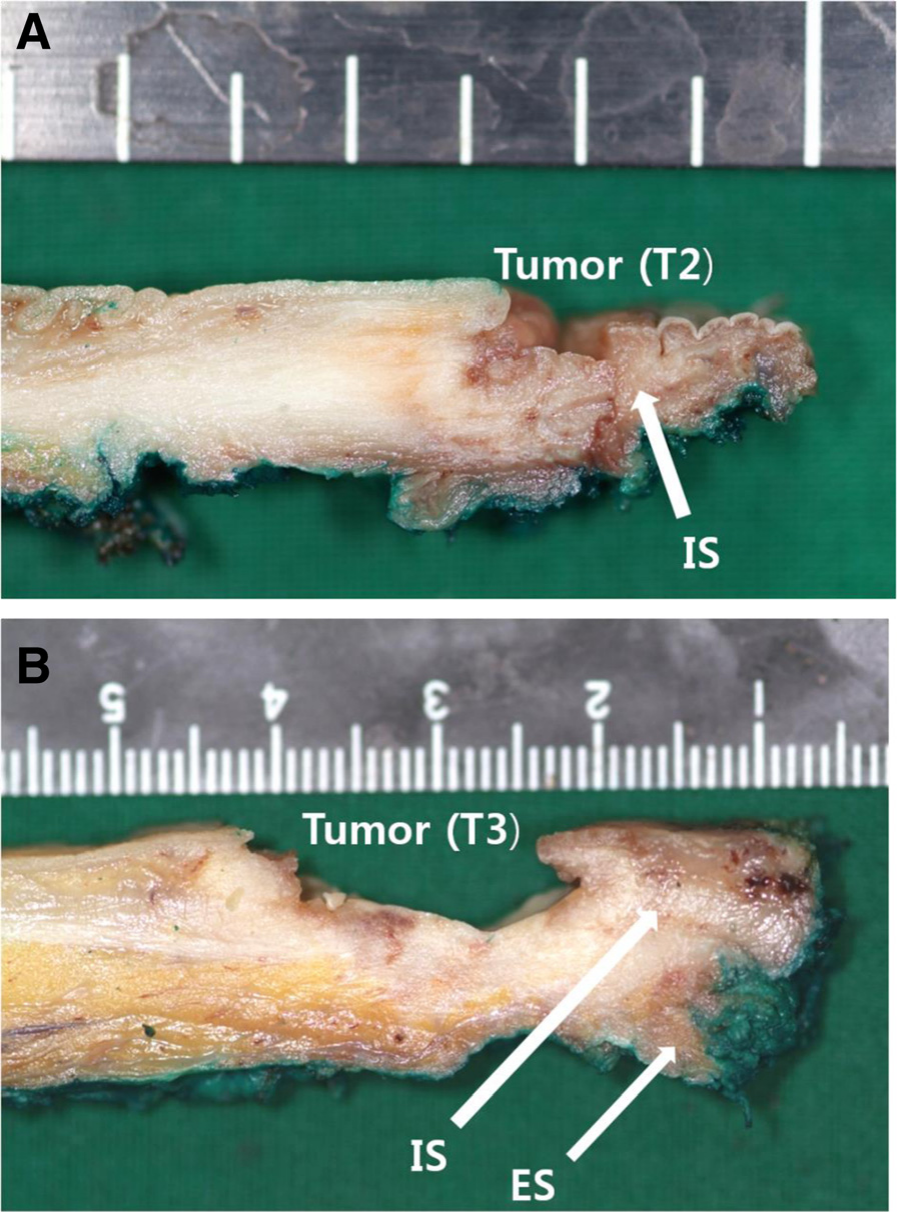
Macroscopic finding of the comparison between T2 and T3 tumor. **a** standard ISR has enough distal and lateral surgical margins in case of T2 tumor. **b** it is often difficult to ensure a safe resection margin in case of T3 tumor. The resected specimen of extended ISR in T3 tumor shows enough surgical margin included upper part of ES. IS internal sphincter. ES external sphincter

After pelvic dissection with an abdominal approach, intersphincteric resection was performed by perineal approach. For T2 (Group I) lesions, standard ISR was performed. After an incision was placed on the intersphincteric groove level, the gap between the internal and external sphincters was incised and dissection continued along the levator ani in the intersphincteric plane to connect with the pelvic dissection from the abdominal approach. In this process, the intersphincteric plane was dissected while the pelvis was illuminated from the abdomen (Fig. 1a). For the patient with T3 (Group II) lesions, extended ISR was performed. To ensure the safe radial resection margin after dissecting the anal sphincter plane, the deep part of the external sphincter was incised and removed, superficial part and subcutaneous part was preserved at least (Fig. 1b). Macroscopic findings of the resected specimen in group I and II are shown in Fig. 2.

Then, a colonic J-pouch - anal anastomosis was performed with 3–0 Vicryl sutures. In this procedure, 3-point interrupted sutures were performed, which the cut edge of the J-pouch was anastomosed to the cut edge of the anoderm connecting fixed levator ani muscle, to invaginate the anastomotic area and prevent the prolapse of the colon. Temporal diverting ileostomy was carried out for fecal detour, and then, the abdomen was closed. Unless there were special circumstances, ileostomy closure was performed in 6 months after surgery.

### Follow-up

During the first 6 months after surgery, outpatient follow-up was carried out every 3 months. Clinical symptoms were observed during the follow-up interview and a clinical laboratory test (CBC and CEA) and a radiographic examination (chest radiography) were conducted. In 6 months postoperatively, abdominal CT and colonoscopy were performed.

The patients with restored ileostomy were followed using a standard protocol every 6 months for 5 years. The assessment of defecatory function was evaluated through personal interviews about stool frequency in a 24-h period and incontinence status assessed by Kirwan’s classification [13] and Wexner’s score [14] at the 12^th^ to 24^th^ month after ileostomy closure.

To evaluate local recurrence anddistant metastasis, abdominal computed tomography (CT) every 6 months for 2 years, and then once a year and colonoscopy were performed every 1 years. Anorectal manometry was not routinely performed to evaluate postoperative sphincter function.

### Statistical analysis

Statistical analysis was performed using MedCalc Statistical Software version 14.10.2 (MedCalc Software bvba, Ostend, Belgium). Differences in distribution were calculated using the *t*-test for continuous variables, and chi-square test or Fisher’s exact test for categorical variables. Overall survival (OS) rates were analysed using the Kaplan–Meier method, and statistical significance of the survival rates was evaluated using the log-rank test. A *P*-value of < 0.05 was considered statistically significant.

### Ethics statement

The study protocol was approved by the Institutional Review Board of Pusan National University Hospital (Reference No.: PNUH IRB E-2015122). Written informed consent forms concerning this procedure were obtained for all patients.

## Results

Patients and tumor characteristics are shown in Table 1. Of a total of 62 patients, 24 patients underwent standard ISR (group I; mean age 56.8 years, 15 males and 9 females), and 38 patients had extended ISR (group II; mean age 57.3 years, 23 males and 15 females).

**Table 1:** Patient and tumor characteristics

	Group I	Group II	*P* value
No. of patients	24	38	
Mean age (yr)^a^	56.8 (10.4)	57.3 (11.7)	
Gender			
Male Female	15 9	23 15	
Distalsurgicalmargin (cm)^a^	1.69 (0.12)	1.58 (0.15)	0.056
Lateral surgical margin	0 (0)	0 (0)	0.87
< 1 mm 1-3 mm > 3 mm	5 (20.9) 19 (79.1)	6 (15.8) 32 (84.2)	
yTNM stage			<0.05
Stage I Stage II Stage III	19 (79.2) 0 (0) 5 (20.8)	0 (0) 22 (57.9) 16 (42.1)	

^a^Data are mean (SD)

Data are presented as number or number (%)

### Morbidity and mortality

Patients who had undergone operations were found to have early complications, such as temporary urinary retention (group I; 10 cases: group II; 15 cases), postoperative paralytic ileus (group I; 2 cases: group II; 3 cases), perineal abscess (group I; 1 case: group II; 1 case), and anastomotic leakage (group I; 1case: group II; 1 case). The patients who developed a perineal abscess healed with incision and drainage. The patients who developed an anastomotic leakage healed with conservative treatment (antibiotic therapy and TPN) without surgical intervention.

Late complications, such as anastomotic stricture (group I; 6 cases: group II; 10 cases), rectovaginal fistula (group I; 0case: group II; 1 case) after stoma closure were observed (Table 2). The patients who developed anastomotic stricture, however, the degree of narrowing was not severe, and most patients improved after laxative administration. Two patients recovered with endoscopic balloon dilatation. One patient developed a rectovaginal fistula, which was endoscopically closed with fibrin glue injection. There was no postoperative mortality in both groups.

**Table 2:** Postoperative complications

	Group I (*N* = 24)	GroupII(*N* = 38)	*P* value
Early complications			
Temporary urinary retention	10 (41.7)	15 (39.5)	0.92
Postoperative paralytic ileus	2 (8.3)	3 (7.9)	0.68
Perineal abscess	1 (4.0)	1 (2.6)	0.62
Anastomotic leakage	1 (4.0)	1 (2.6)	0.62
Late complications			
Anastomotic stricture	6 (25.0)	10 (26.3)	0.85
Rectovaginal fistula	0	1 (2.6)	

Data are presented as number (%)

### Oncologic results

All patients received potentially curative R0 ISR. The average distance measured to the distal resection margin during surgery was 1.69 cm in group I and 1.58 cm in group II. The lateral resection margin was negative in all cases. For the distance from the tumor to the resection margin, 5 cases were 1–3 mm in group I and 5 cases were the same in group II. The distance was greater than 3 mm in 19 cases in group I and 32 cases in group II (Table 1).

In postoperative pathologic tumor stages were as follows: group I (ypT2N0, *n* = 19; ypT3N0, *n* = 1; ypT2N1, *n* = 4), group II (ypT2N0, *n* = 2; ypT3N0, *n* = 26; ypT3N1, *n* = 7; ypT3N2, *n* = 3) (Table 3). In group I, one patient corresponded to T2 in the preoperative clinical stage was confirmed to be T3 after postoperative pathologic examination. Conversely, clinicopathological downstaging was observed by neoadjuvant chemoradiotherapy; two patients were downstaged to be T2 from T3 after pathological examination in group II. One case was observed from stage III to stage I in group I and 6 cases were observed from stage III to stage II in group II (Tables 4 and 5).

**Table 3:** Differences between preoperative yTN and postoperative ypTN staging

	Preoperative (yTN)		Postoperative (ypTN)	
	Group I	Group II	Group I	Group II
Stage I				
T2N0	19	0	19	2
Stage II				
T3N0	0	22	1	26
Stage III				
T2or3N1-2	5	16	4	10

Data are presented as number

**Table 4:** Preoperative yT vs. postoperative ypT classification

	ypT1	ypT2	ypT3	Total
yT2	-	23	1^a^	24 (38.7)
yT3	-	2	36	38 (62.3)
Total	-	25 (40.3)	37 (59.7)	62 (100)

Data are presented as number or number (%)

^a^Patient overstaged from T2 to T3 in group I

yT: clinical T stage after neoadjuvant treatment

ypT: histological T stage after neoadjuvant treatment

**Table 5:** Preoperative yN vs. postoperativeypN classification

	ypN0	ypN1	ypN2	Total
yN0	41	-	-	41 (66.1)
yN+	7	11	3	21 (33.9)
Total	48 (77.4)	11 (17.8)	3 (4.8)	62 (100)

Data are presented as number or number (%)

yN: clinical N stage after neoadjuvant treatment

ypN: histological N stage after neoadjuvant treatment

During the median follow-up period of 115 (range, 34–172) months after surgery, two patients with local recurrence and six patients with distant metastasis were observed. For distant metastasis, 2 cases of liver metastasis occurred in group I and 3 cases of liver metastasis and 1 case of both liver metastasis and lung metastasis occurred in group II.

**Fig. 3:**
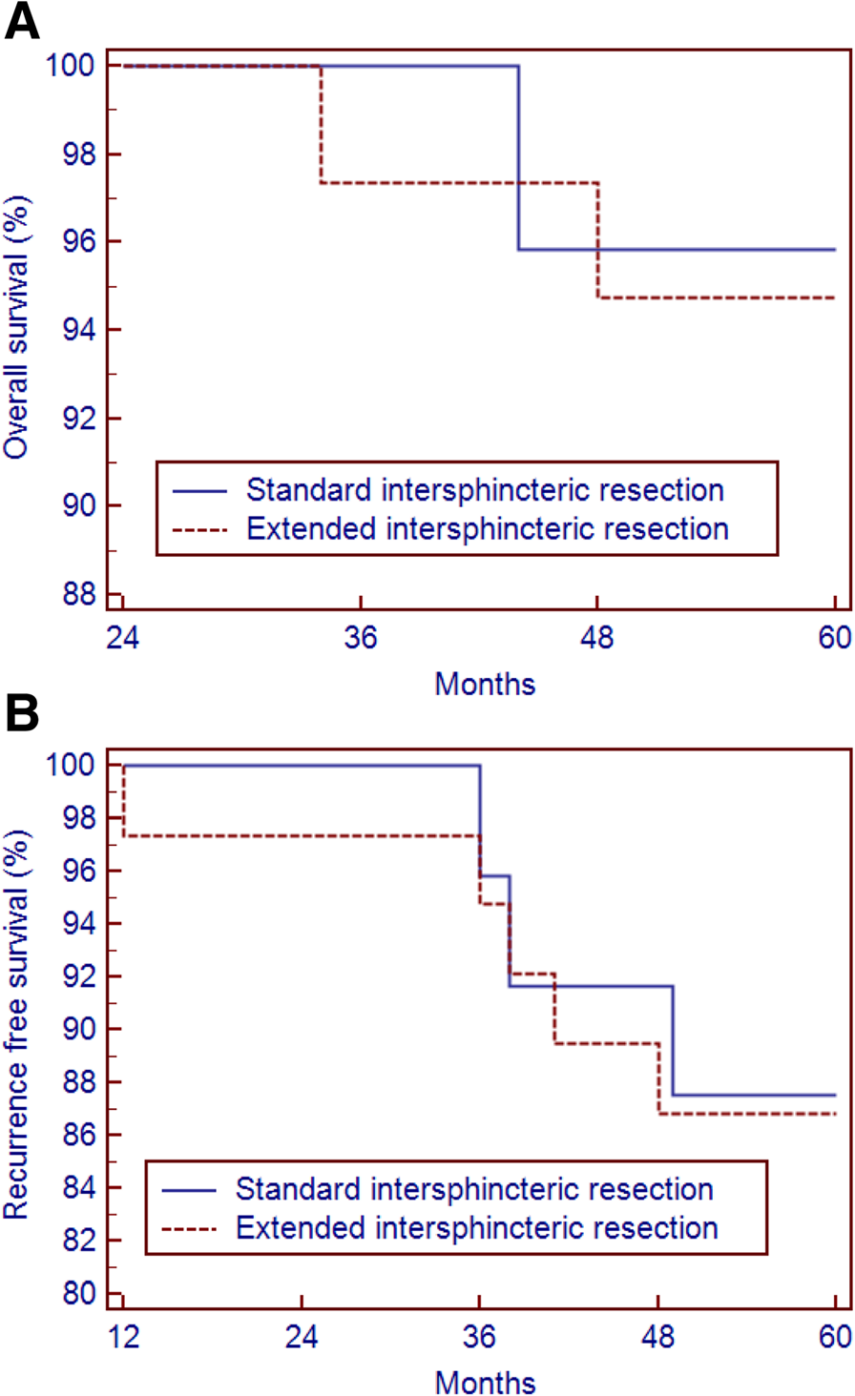
**a** Five-year overall survival rates were 95.8 % for standard ISR and 94.7 % for extended ISR. **b** Five-year recurrence-free survival rates were 87.5 % for standard ISR and 86.8 % for extended ISR

Five-year overall survival (OS) and recurrence-free survival were 95.8 % and 87.5 %, respectively, in group I and 94.7 % and 86.8 %, respectively, in group II (Fig. 3a and b).

### Functional results

Bowel functions were evaluated and compared at the 12^th^ and 24^th^ months after ileostomy closure in both groups. The mean frequency of defecation per day was 3.54 times in group I and 4.29 times in group II at the 12^th^ month (*p* < 0.05). At the 24^th^ month, the mean frequency was decreased in group I by 2.21 times and in group II by 2.39 times on average, demonstrating no significant difference between both groups. All patients who had more than three instances of defecation per day showed stool fragmentation and four patients with two instances of defecation also showed the same symptoms.

In the comparison based on Kirwan’s classification, group I showed grade I continence (perfect continence) in 14 patients, grade II continence (flatus incontinence: not refraining from flatulence or mucus soiling) in six patients, grade III continence (minor soiling: incontinence 1–2 times a week) in three patients, and grade IV continence (major soiling: almost daily incontinence) in one patient. Group II exhibited grade I continence in 22 patients, grade II continence in ten patients, grade III continence in three patients, and grade IV continence in three patients at the 12^th^ month. No patient exhibited grade V continence. After 24 months, group I showed grade I continence in 19 patients, grade II continence in three patients, grade III continence in one patient, and grade IV continence in one patient. Group II exhibited grade I continence in 25 patients, grade II continence in eight patients, grade III continence in three patients, and grade IV continence in two patients. In this comparison, the two groups showed no statistically significant difference at the 12^th^ and 24^th^ months. The comparison of Wexner score of both groups revealed that at the 12^th^ month, the average score was 7.33 ± 2.8 in group I and 8.18 ± 2.9 in group II, and at the 24^th^ month, the average score was 5.21 ± 1.7 in group I and 5.82 ± 1.9 in group II. There was no statistically significant difference between the two groups (Table 6).

**Table 6:** Functional results at different times after stoma closure (12 months, 24 months)

	12 months			24 months		
	Group I	Group II	*P* value	Group I	Group II	*P* value
Stoolfrequency (per day)^a^	3.54 (1.38)	4.29 (1.46)	<0.05	2.21 (1.03)	2.39 (1.12)	0.31
Kirwan classification^b^			0.86			0.91
I	14	22		19	25	
II	6	10		3	8	
III	3	3		1	3	
IV	1	3		1	2	
V	o	0		0	0	
Wexner score^c^	7.33 (2.84)	8.18 (2.91)	0.26	5.21 (1.67)	5.82 (1.93)	0.21

^a^Data are presented as number (SD)

^b^Data are presented as number: Grade I = perfect; Grade II = incontinence of flatus; Grade III = occasional minor soiling; Grade IV = frequent major soiling; Grade V = incontinence

^c^Data are presented as number (SD): 0 = perfect continence; 20 = major incontinence

## Discussion

For the curative resection of very low rectal cancer within 4 cm from the anal verge, abdominoperineal resection is accepted for standard surgical procedure. However, this procedure is inevitably accompanied by a permanent stoma, compromising quality of life due to psychological and social limitations. Moreover, the conventional low anterior resection (LAR) might not guarantee a safe surgical margin in some cases with very low rectal cancer. These surgical and oncologic backgrounds stimulated surgeons to devise other surgical modifications of sphincter-preserving procedures. In recent years, ISR with coloanal anastomosis has been proposed to avoid permanent colostomy for very low rectal cancers and was reported in many studies [5–10].

Traditional standard ISR refers to the extension of rectal resection into the intersphincteric plane with removal of the internal sphincter and restoring bowel continuity by coloanal anastomosis. Although it is technically difficult, ISR is associated with morbidities and mortalities similar to those in with low anterior resection and APR. Schiessel et al. reported treatment outcomes of 38 patients by applying ISR for the first time in 1994 [8]. Oncologic and functional tests conducted in 121 patients for 16 years reported that ISR has no difference in survival or recurrence rates compared to low anterior resection or APR [15]. Gamagami et al. reported excellent results of ISR compared to APR in low rectal cancer patients with a 7.9 % local recurrence rate and 78 % 5-year survival [16]. Saito et al. reported a local recurrence rate of 5.3 % and 5-year overall survival and disease-free rates of 91.9 % and 83.2 %, respectively, in patients with rectal cancer located within 5 cm of the anal verge [17].

In more recent years, Chamlou et al. reported a local recurrence rate of 6.6 %, a 5-year overall survival rate of 82 %, and a disease-free survival rate of 75 % in patients with rectal cancer located within 3.5 cm from the anal verge [18]. These results demonstrate the pathologic appropriateness of ISR during the surgical treatment of very low rectal cancers. These improved results induced downstaging due to neoadjuvant chemoradiotherapy, more accurate diagnosis of preoperative stage with magnetic resonance imaging and endoanal ultrasonography, and technical improvements in surgery.

However, in cases with T3 or in tumors that invaded the internal sphincter even after chemoradiation therapy, it is often difficult to complete curative resection. We examined the 5-year overall survival and recurrence-free survival rate, the local recurrence, and the distant metastasis by performing extended ISR with quadrant resection of the external sphincter. The 5-year overall survival and the disease-free survival were 95.8 % and 87.5 %, respectively, in group I, and 94.7 % and 86.8 %, respectively, in group II. There were two patients with local recurrence and six patients with distant metastasis. This suggests no significant difference in survival and recurrence rates in both groups with satisfactory values in the comparison of results of a standard ISR in the previous studies.

To apply this surgical technique to patients, accurate preoperative diagnosis is important. For this diagnosis, magnetic resonance imaging or endoanal ultrasonography was conducted to estimate the tumor-free circumferential and distal margin. Based on these results, patients with the invasion of the external sphincter and anal levator were excluded. Several studies in recent years have reported results that showed that preoperative magnetic resonance imaging (MRI) is very helpful and accurate in identifying the invasiveness of tumors. Urban et al. reported 98 % specificity and 100 % sensitivity in the accuracy of magnetic resonance imaging in predicting the invasiveness of tumors [19]. However, they also reported that preoperative chemoradiotherapy somewhat reduces the accuracy due to the fibrotic changes of the tumor. Performing extended ISR procedures without accurate information on whether the sphincter is involved may cause local recurrences and frequent postoperative complications.

The principal disadvantage of ISR is the risk of a poor functional outcome. If the sphincter is removed and rectal storage capability is lost, functional bowel disorders, such as fecal urgency, frequency, stool fragmentation and fecal incontinence, may occur. Previous studies have reported normal continence (Kirwan grade 1) rates of 29 to 86.3 % and major incontinence (Kirwan grade 4) rates of 0 to 25.8 % after ISR [15, 17, 18, 20–22]. Physiological mechanisms that maintain continence are influenced by a number of factors, such as the high pressure zone (HPZ) by the adjustment of the sphincter, organized movement between the anorectal angle and pelvic floor muscles, and the anorectal sensation and reflex. The principal mechanism that controls continence is the maintenance of the resting pressure that approximately 85 % is maintained by internal sphincter tone and the rest is influenced by the external sphincter. The average resting pressure is approximately 90 cmH_2_O, is lower in women than men, and decreases with age. Kohler et al. reported that partial ISR reduces the pressure from 105 cmH_2_O to 75 cm H_2_O and total ISR reduces it up to 40 cm H_2_O, and the decrease of resting pressure depended on the extent of the resection of the internal sphincter [23].

In this study, the upper quadrant of the external sphincter was also excised with T3 lesions. Thus, the maintenance of continence was a significant issue. It is important for the function of the preserved external sphincter to secure the precise intersphincteric plane dissection and apply atraumatic techniques. We used an illuminator as an assistant to illuminate the pelvis for precise dissection. We also applied an anal retractor (the Lone Star Retractor® (Lone Star Medical Products Inc.)) to prevent trauma to the external sphincter and anoderm and to secure sufficient space for the colonic J-pouch anal anastomosis during the transanal approach. In this study, both groups showed no statistically significant difference in 12 months and 24 months for the Wexner score and the Kirwan grade.

Various types of colonic pouches have been widely used to increase the reservoir capacity of the neorectum in treatments involving anastomosis following ISR for very low rectal cancer. Parc et al. [24] and Hallbook et al. [25] designed a J-shaped colonic pouch to reduce the severity of functional problems related to defecation compared to straight anastomosis. Subsequent studies concluded that anastomosis using a colonic J-pouch could reduce the frequency of defecation and laxative use [26, 27]. On the other hand, Schiessel et al. reported that the effect of the colonic reservoir improved by the pouch lasts for only the first 3 months after surgery and that anasotomosis of a pouch down to the level of the dentate line is technically difficult due to the bulk of mesocolic fat and the length of the anal canal [15]. However, bowel function may be improved by decreased bowel motility, and anastomotic leakage may be reduced by better blood supply to the anastomosis site and decreased ‘dead space’ in the pelvic cavity [28–31]. Thus, it should be the preferred technique if it is practicable.

## Conclusion

Acceptable oncologic and functional outcomes were obtained by using extended ISR in patients with very low rectal cancer. This study provides evidence that extended ISR including quadrant resection of upper external sphincter in case of T3 low rectal cancer is also seemed to be acceptable procedure comparing with standard ISR in T2 low rectal cancer with suitable patient selection through the appropriate preoperative evaluation.

## Ethics approval and consent to participate

The study protocol was approved by the Institutional Review Board of Pusan National University Hospital (Reference No.: PNUH IRB E-2015122). Written informed consent forms concerning this procedure were obtained for all patients.

## Consent for publication

We obtained written consent to publish all the personal details included in our dataset from all participants prior to surgery.

## Availability of data and materials

All datasets on which the conclusions of the manuscript rely was provided as additional supporting file.

## Abbreviations

APR: abdominoperineal resection
ISR: Intersphinctericresection
LARC: locally advanced rectal cancer
TME: total mesorectal excision
